# Real-world characterization of vestibular contributions during locomotion

**DOI:** 10.3389/fnhum.2023.1329097

**Published:** 2024-01-08

**Authors:** Liam H. Foulger, Jesse M. Charlton, Jean-Sébastien Blouin

**Affiliations:** ^1^School of Kinesiology, University of British Columbia, Vancouver, BC, Canada; ^2^School of Biomedical Engineering, University of British Columbia, Vancouver, BC, Canada; ^3^Institute for Computing, Information and Cognitive Systems, University of British Columbia, Vancouver, BC, Canada; ^4^Djavad Mowafaghian Centre for Brain Health, University of British Columbia, Vancouver, BC, Canada

**Keywords:** wearable sensors, vestibular stimulation, locomotion, balance, real-world, inertial measurement units

## Abstract

**Introduction:**

The vestibular system, which encodes our head movement in space, plays an important role in maintaining our balance as we navigate the environment. While in-laboratory research demonstrates that the vestibular system exerts a context-dependent influence on the control of balance during locomotion, differences in whole-body and head kinematics between indoor treadmill and real-world locomotion challenge the generalizability of these findings. Thus, the goal of this study was to characterize vestibular-evoked balance responses in the real world using a fully portable system.

**Methods:**

While experiencing stochastic electrical vestibular stimulation (0–20 Hz, amplitude peak ± 4.5 mA, root mean square 1.25 mA) and wearing inertial measurement units (IMUs) on the head, low back, and ankles, 10 participants walked outside at 52 steps/minute (∼0.4 m/s) and 78 steps/minute (∼0.8 m/s). We calculated time-dependent coherence (a measure of correlation in the frequency domain) between the applied stimulus and the mediolateral back, right ankle, and left ankle linear accelerations to infer the vestibular control of balance during locomotion.

**Results:**

In all participants, we observed vestibular-evoked balance responses. These responses exhibited phasic modulation across the stride cycle, peaking during the middle of the single-leg stance in the back and during the stance phase for the ankles. Coherence decreased with increasing locomotor cadence and speed, as observed in both bootstrapped coherence differences (*p* < 0.01) and peak coherence (low back: 0.23 ± 0.07 vs. 0.16 ± 0.14, *p* = 0.021; right ankle: 0.38 ± 0.12 vs. 0.25 ± 0.10, *p* < 0.001; left ankle: 0.33 ± 0.09 vs. 0.21 ± 0.09, *p* < 0.001).

**Discussion:**

These results replicate previous in-laboratory studies, thus providing further insight into the vestibular control of balance during naturalistic movements and validating the use of this portable system as a method to characterize real-world vestibular responses. This study will help support future work that seeks to better understand how the vestibular system contributes to balance in variable real-world environments.

## 1 Introduction

Maintaining an upright posture as we navigate the world is crucial for performing many activities of daily life. Bipedal locomotion requires us to move our centre of mass outside the base of support in a “controlled falling” manner, resulting in instability mainly in the mediolateral (ML) direction as the passive dynamics of the legs provide stability for the anteroposterior (AP) direction (McGeer, 1990; Perry, 1992; Winter, 1995). Consequently, bipedal locomotion requires an active feedback control of balance that involves the integration of multisensory information (Bauby and Kuo, 2000). One source of sensory information contributing to balance control arises from the vestibular system which encodes head movement in space and drives whole-body balance responses (Day and Fitzpatrick, 2005a). Electrical vestibular stimulation (EVS) is a common method to probe vestibular balance responses in humans that involves applying a small current through surface electrodes placed on the mastoid processes (Fitzpatrick and Day, 2004). This current activates all vestibular afferents (Kwan et al., 2019; Forbes et al., 2023) and introduces an error signal of vestibular origin that evokes stereotyped muscle, kinetic, and kinematic balance responses (Nashner and Wolfson, 1974; Bent et al., 2004; Fitzpatrick and Day, 2004; Dakin et al., 2010). Using this approach, researchers have demonstrated that the vestibular control of balance during locomotion decreases with increases in locomotor speed and step cadence (Jahn et al., 2000; Dakin et al., 2013; Dietrich et al., 2020), findings that corroborate clinical observations (Brandt et al., 1999). Furthermore, the vestibular control of balance during locomotion exhibits phasic modulation depending on when lower limbs contact the ground and the role of muscles in balance control. Specifically, lower limb muscle responses to EVS occur mainly during the stance phase of the ipsilateral limb (Blouin et al., 2011; Dakin et al., 2013) and whole-body balance responses, measured from ground reaction forces and centre of pressure displacement, occur at the midpoints between heel strikes (Dietrich et al., 2020; Magnani et al., 2021). One proposed model suggests that head kinematic variability drives this modulation in vestibular control of balance during locomotion (MacNeilage and Glasauer, 2017; Dietrich et al., 2020).

While the vestibular control of balance has been well characterized within the research laboratory, there are differences between indoor treadmill and real-world locomotion that must be examined (Schmitt et al., 2021). For example, the natural variability of head kinematics exhibits smaller vertical head translation on treadmill compared to overground walking (Bloomberg et al., 1992; Hirasaki et al., 1993, 1999). Given that head kinematic variability may play a role in modulating the vestibular control of locomotion, quantifying head kinematics and vestibular-evoked responses in variable and dynamic contexts encountered in daily activities is critical. Optic flow cues also differ between overground and treadmill walking; treadmill walking potentially introduces a visual-vestibular sensory mismatch that could alter sensorimotor integration mechanisms underlying the control of balance during locomotion (Deshpande and Patla, 2005). Given that deviations in optic flow can increase variability in balance control (Pickhinke et al., 2014), it will be important to maintain naturalistic optic flow when quantifying vestibular-evoked balance responses during locomotion. Thus, we need to develop methods and analyses to probe vestibular-evoked responses during real-world locomotion to ultimately understand the neural mechanisms underlying the feedback control of balance during human locomotion.

In this study, we developed a fully portable system integrating inertial measurement units (IMUs) and EVS to (1) determine if we could characterize the vestibular control of balance during locomotion in the real-world and identify phase-dependent modulations reported in previous in-laboratory experiments and (2) compare the decrease in vestibular-evoked responses at faster cadences (78 steps/min at ∼0.8 m/s and 52 steps/min at ∼0.4 m/s) with previously established in-laboratory changes at the same cadences and speeds (Dakin et al., 2013; Dietrich et al., 2020). A secondary objective was to determine the appropriate number of strides needed to characterize EVS-evoked vestibular responses in the real-world. In accordance with previous reports, we predicted that using our portable, IMU-based system, we would observe lower limb vestibular response peaks during the ipsilateral stance phase and whole-body vestibular response peaks bilaterally during the middle of the single limb support phases. Secondly, we hypothesized that the magnitude of the vestibular-evoked balance responses would decrease with increasing cadence and speed.

## 2 Materials and methods

### 2.1 Participants

We recruited ten adult participants (5 female and 5 male, age 26 ± 3 years, height 170 ± 12 cm, mass 75 ± 24 kg) with no neurological or musculoskeletal impairments. A power analysis given an effect size of 1.32 [via *F*_1,9_ = 15.5 from vestibular balance responses using centre of pressure displacement (Dietrich et al., 2020)] and a desired α = 0.05 and power = 0.95 for a one-sided paired sample *t*-test yielded a minimum sample size of eight participants. The outcome measure from this previous study (centre of pressure displacement) is different from our study (kinematics); however, these variables are closely linked so it was likely that the responses would be similar in magnitude. We added two additional participants to account for the possibility of increased noise due to the real-world nature of this experiment. This experiment was reviewed by the University of British Columbia’s Clinical Research Ethics Board (H22-01776) and participants provided written informed consent prior to enrolling in the study.

### 2.2 Setup

**FIGURE 1:**
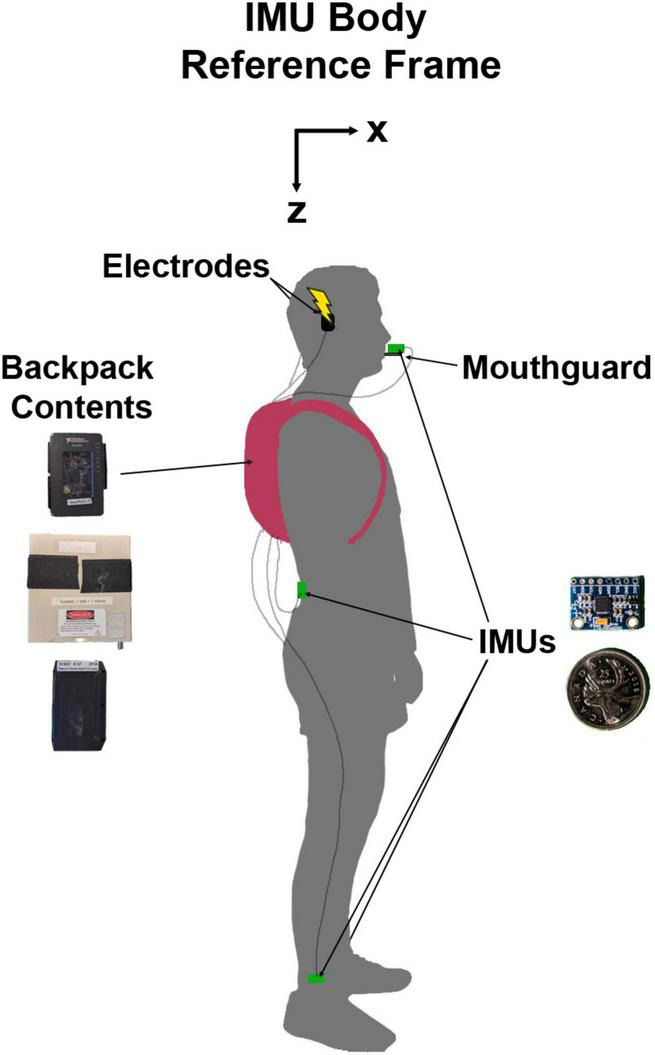
Diagram of experimental set-up with a participant in N-pose. The inertial measurement units (IMUs) and their protective backings were 19.8 mm wide, 15.1 mm tall, and 4.2 mm thick. The backpack contained the myRIO-1900 **(top)**, stimulator **(middle)**, and battery **(bottom)** which combined weighed approximately 1.8 kg. The IMU body reference frame was not a global reference frame. Here, it is presented as oriented during the participant in N-pose but would move with the local body segment orientation.

We attached four IMUs (MPU 6050; accelerometer range = ±16 g; gyroscope range = ±1000 °/s) to the participant for the duration of the experiment (Figure 1). The first was attached to a custom-made mouthguard using a vinyl polyciliate dental impression and molded using ethylene vinyl acetate. The IMU was mounted on a small cast acrylic tab attached to the mouthguard so that it sat outside the mouth. This allowed for direct measurement of head kinematics without skin motion artefact (Wu et al., 2016). The second was placed on the low back over the third lumbar spinous process. The height of this sensor was measured for each participant (1.08 ± 0.11 m SD). This location was chosen to approximate the body centre of mass and therefore estimate the resultant vestibular-evoked whole-body balance responses, as centre of mass acceleration is related to horizontal ground reaction forces which have been used to characterize vestibular responses during locomotion in previous studies (Hannan et al., 2021; Magnani et al., 2021). Other authors have also positioned IMUs on the lumbar vertebrae to extract gait features and estimate centre of mass displacement, acceleration, and ground reaction forces (McCamley et al., 2012; Howell et al., 2015; Del Din et al., 2016; Storm et al., 2016; Cardarelli et al., 2020). The final two IMUs were placed just above the lateral malleoli of the right and left ankles to detect gait events (heel strike and toe-off) (Bötzel et al., 2016) and characterize vestibular-evoked responses in the lower limbs (de Melker Worms et al., 2017). The IMUs were fixed to the skin using single- and double-sided hypoallergenic tape.

### 2.3 Sensor calibration

Prior to data collection, we calibrated each IMU to correct the gain of the accelerometers and any offsets in the accelerometers and gyroscopes. Once attached to the participants, we calibrated the IMUs to a standard body reference frame of X: forward, Y: right, and Z: down by recording two static poses for each IMU (Chen et al., 2022). To define the Z axis as pointing downwards, the participants stood upright (N-pose; Figure 1; Robert-Lachaine et al., 2017) with their head pitched so that Reid’s plane was perpendicular to gravity. Reid’s plane was chosen for the calibration pose so that we could orient the head with respect to the net response evoked by EVS and provide feedback to participants to maximize the vestibular-evoked responses (see section “2.6 Protocol” below). For the second pose, participants pitched each respective body segment ∼90 degrees forward and down to define an approximate X axis facing forward. To determine the approximate X axis and true Z axis, the axes were defined as opposite to the net acceleration vector caused by the gravitational field. The Y axis was defined as the cross product of the Z-axis and the approximate X-axis, and the true X axis was determined by the cross product of the Y and Z axes.

### 2.4 Stimulus

Binaural bipolar electrical vestibular stimulation was applied to the participants via gel-coated carbon rubber electrodes (9 cm^2^) taped to the mastoid processes. We delivered the electrical vestibular stimuli as stochastic waveforms (stochastic EVS; 0–20 Hz, amplitude peak ± 4.5 mA, root mean square 1.25 mA) created using LabVIEW 2019b (National Instruments, TX, United States) and delivered with a constant current isolated linear stimulator (STIMSOLA, Biopac Systems Inc., CA, United States). The amplitude of the stimulus was chosen to replicate previous studies that have successfully evoked vestibular balance responses (Blouin et al., 2011; Dakin et al., 2013) and the bandwidth was reduced to 0–20 Hz given that we expected kinematic responses to occur below 10 Hz, based on previous observations in EVS-evoked centre of pressure responses (Dietrich et al., 2020). EVS activates the primary otolith and semicircular canal vestibular afferents (Goldberg et al., 1982; Kwan et al., 2019; Forbes et al., 2023) which generates a virtual angular velocity signal around an axis pointing ∼17–19 degrees up from Reid’s plane (Schneider et al., 2002; Fitzpatrick and Day, 2004; Chen et al., 2020) and an inferred linear acceleration signal that is dependent on the orientation of the head (Khosravi-Hashemi et al., 2019). These signals are craniocentric, meaning that the direction of the responses are dependent on head position (Lund and Broberg, 1983; Mian and Day, 2014). We chose stochastic EVS as the stimulus because it allows for the characterization of phasic vestibular responses over the stride cycle with reduced testing time and minimized disruptions of the gait pattern compared to square wave stimuli (Blouin et al., 2011).

### 2.5 Portable System

To simultaneously measure kinematics using the IMUs and deliver the stochastic EVS outside of the laboratory, we developed a portable system using a reconfigurable I/O device (myRIO-1900, National Instruments) with a field-programmable gate-array, running LabVIEW 2019b (National Instruments). The microcontroller communicated to each of the IMUs with I^2^C protocol via a wired connection and an analog output was connected to the constant current stimulator to drive the stochastic EVS. The stochastic EVS was sent from, and the data were saved directly to the myRIO-1900 at a rate of 200 Hz. We powered both the myRIO-1900 and the stimulator with a 12 V battery (TalentCell, China). We placed the myRIO-1900, constant current stimulator and battery in a small backpack worn by the participant. This weighed approximately 1.8 kg. Finally, a laptop (ThinkPad X1 Nano, Lenovo, China) wirelessly communicated with the myRIO-1900 to allow for system control and data visualization.

### 2.6 Protocol

To replicate previous work that characterized vestibular responses with electromyography, ground reaction forces, and centre of pressure displacement (Iles et al., 2007; Blouin et al., 2011; Dakin et al., 2013; Dietrich et al., 2020; Magnani et al., 2021), participants walked outside on pavement at cadences of 52 steps/min (0.37 ± 0.02 m/s SD) and 78 steps/min (0.82 ± 0.10 m/s SD). Cadences were guided by a metronome played to the participants using earbud headphones. We instructed participants to walk at the step lengths required to reach gait speeds of 0.4 and 0.8 m/s, and we provided ongoing feedback based on visual monitoring of foot placement and whether the participant reached the expected distance travelled at the end of each trial. We determined the distance travelled from start to end of each trial using global positioning system measurements from a smartphone app (phyphox, RWTH Aachen University, Germany) to estimate average gait speed. During the trials, we instructed participants to keep their head pitched up at ∼17–19°, to maximize the net responses evoked by EVS (Fitzpatrick and Day, 2004). We monitored head pitch using the orientation calculated from the mouthguard IMU and provided verbal feedback for participants to maintain this head posture. Participants completed three trials of 200 s at the 52 steps/min cadence and three trials of 135 s at the 78 steps/min cadence. Overall, this resulted in a minimum of 250 strides performed per condition for each participant, given this has previously been identified as the minimum number of strides required to minimize time-dependent coherence-related error during locomotion (Blouin et al., 2011). We provided rest breaks to participants whenever necessary to prevent any fatigue.

### 2.7 Signal analysis

After data collection, the signals were linearly interpolated to correct for any possible missed samples during data recording. On average, 7.4 samples were dropped each trial (range = 0–31 samples), which is approximately one sample dropped for every 4500 samples, or 22.5 s. Following the post-processing and calibration, the IMU data were filtered with a dual pass lowpass fourth order Butterworth filter at 80 Hz. The stochastic EVS data were low pass filtered at 20 Hz with a dual pass fourth order Butterworth filter.

#### 2.7.1 IMU orientation estimation

To determine the orientation of the various body segments, we calculated the tilt orientation of the IMUs in the pitch (around Y-axis) and roll (around X-axis) axes with a complementary filter (Gui et al., 2015):

$$\theta_{i}=\left(\theta_{i-1}+\omega_{i}*d\,t\right)\,G+\left(\varphi_{i}\right)\,\left(1-G\right)$$

where θ_*i*_ is the *i*th sample point of the orientation estimate with respect to vertical in the one axis, ω is the angular velocity around the same axis from the gyroscope, *dt* is the time step between samples (5 ms in our study), *G* is the weighting factor between the gyroscope and accelerometer estimates which was set to 0.995, and φ is the orientation estimate in the same axis from the accelerometer data. If the net acceleration was 10% above or below 9.81 m/s^2^, only the gyroscope information was used to estimate the orientation at that given sample point. Below are the equations for calculating the accelerometer orientation estimates in pitch and roll, respectively, given the IMU body reference frame used in this study and the accelerations recorded from the IMUs ($\text{a}=\left[\ddot{x}\,\ddot{y}\,\ddot{z}\right]$).

$$p\,i\,t\,c\,h=\varphi_{i}=a\,t\,a\,n\,2\,d\,\left({\ddot{x}}_{i},{\ddot{z}}_{i}\right)$$

$$r\,o\,l\,l=\varphi_{i}=-a\,t\,a\,n\,2\,d\,\left({\ddot{y}}_{i},{\ddot{z}}_{i}\right)$$

#### 2.7.2 IMU linear acceleration gravity correction

Linear accelerometers cannot distinguish between gravitational and inertial accelerations. To capture the inertial component of the linear accelerations (a*), the gravitational component must be removed after the recording. Given the estimated IMU orientations from the complementary filter (*R*: rotation matrix, calculated from the pitch and roll orientations and assuming no yaw), we rotated and subtracted the gravitational components (*g* = [00−9.81]) to obtain the corrected inertial component of the linear accelerations (a*). Following the gravity correction, we low-pass filtered the IMU signals at 20 Hz with a dual-pass 4th order Butterworth filter.

$$a^{*}=\left(a-g\,R\right)$$

#### 2.7.3 Head IMU linear acceleration transformation

To properly characterize the head linear accelerations experienced by the vestibular system, we must also remove the linear tangential and centripetal accelerations caused by the radius of rotation from the position of the mouthguard-mounted head IMU to the middle of the head. Although the head does not purely rotate around the vestibular apparatuses, we measured the distance from the mouthguard to the mid-point between the external acoustic meatuses (the approximate location between the vestibular end-organs) and transformed the linear accelerations to account for the difference in location (Blouin et al., 2007; Wu et al., 2016).

$$a_{B}=a_{A}+\alpha \times r_{B-A}+\omega \times \left(\omega \times r_{B-A}\right)$$

Where *a*_*B*_ is the gravity-removed head linear acceleration at the midpoint between vestibular end-organs, *a*_*A*_ is the gravity-removed head linear acceleration at the mouthguard, α is the head angular acceleration, *r*_*B–A*_ is the position vector between the mouthguard and the midpoint between vestibular end-organs, and ω is the head angular velocity.

#### 2.7.4 Stride detection

We divided data into strides by detecting heel strike and toe-off of each limb using the respective ankle IMUs. Heel strike was determined as the local minima of the Y-axis (mediolateral) angular velocity following the large peak at mid-swing while toe-off was estimated as the Y-axis angular velocity zero-crossing prior to the mid-swing peak (Bötzel et al., 2016). We defined a stride as the point of right heel contact to the point immediately before the following right heel contact. We had a provision to remove strides that were either 50% longer or shorter than the participant’s average stride duration, but none met this criterion.

#### 2.7.5 Time-dependent frequency analysis

To determine the phasic modulation of the vestibular-evoked balance responses during locomotion, we calculated the time-dependent coherence, gain, and spectral output power between the EVS and the body kinematics (gravity-corrected ML acceleration from the low back and ankle IMUs, each calculated individually). The roll angular velocities could also be used to determine the vestibular-evoked balance responses; however, we observed smaller and more variable responses, so we did not present these data in the results. Furthermore, the ML linear acceleration at the back is related to the horizontal ground reaction forces which have previously been used to characterize EVS-evoked responses during quiet standing (Dakin et al., 2010) and locomotion (Magnani et al., 2021). Coherence is measure of relatedness across frequencies between an input and output signal, analogous to time-domain correlation analyses, where 0 represents no similarity and 1 represents a perfect match (regardless of scaling) between two signals at a given frequency (Zhan et al., 2006; van Drongelen, 2007). Previous work has shown that the timing between EVS and muscle-evoked responses are consistent throughout the stride-cycle (Blouin et al., 2011; Dakin et al., 2013). Gain represents the scaling between the two signals at a given frequency and the spectral output power represents the magnitude squared of the output signal across frequencies. Given that both coherence and gain are dependent on the spectral output power, this was important to characterize to ensure that changes to coherence and gain are not solely driven by spectral output power changes.

The time-frequency analysis was done using Morlet wavelet decomposition as previously described (Zhan et al., 2006; Blouin et al., 2011; Cohen, 2019). We divided the data into strides and padded each stride with 50% more data from the previous and subsequent strides to avoid distortions in the frequency analysis. To maximize the coherence between the applied EVS and balance responses, we shifted the EVS signal 200 ms forward in time (i.e., delayed) (Dalton et al., 2014; Mian and Day, 2014; Tisserand et al., 2018) prior to the correlation analyses. We performed a Morlet wavelet decomposition with a frequency resolution of 0.5 Hz from 0.5 to 20 Hz to extract the time-dependent cross-spectrum and auto-spectra of the EVS and body segment kinematics. We resampled the data from each stride following this frequency decomposition to avoid distortions in frequency components. For individual participant results, we normalized stride durations and gait event timings (right heel strike, left toe-off, left heel strike, and right toe-off) to the individual participant’s averages. To compare the vestibular-evoked responses between cadence comparisons (see section “2.8 Statistical analysis”), we normalized stride durations and gait event timings to the overall means across participants and cadences. This was to facilitate comparisons across the stride cycle while still anchoring the vestibular responses to the gait events. Then, time-dependent coherence [*C*(τ,*f*)], gain [*G*(τ,*f*)], and body kinematics spectral power [*S*(τ,*f*)] were calculated as follows:

$$C\,\left(\tau ,f\right)=\frac{{\left|P_{x\,y}\,\left(\tau ,f\right)\right|}^{2}}{P_{x\,x}\,\left(\tau ,f\right)\,P_{y\,y}\,\left(\tau ,f\right)}$$

$$G\,\left(\tau ,f\right)=\left|\frac{P_{x\,y}\,\left(\tau ,f\right)}{P_{x\,x}\,\left(\tau ,f\right)}\right|$$

$$S\,\left(\tau ,f\right)={\left|P_{x\,x}\,\left(\tau ,f\right)\right|}^{2}$$

Where τ denotes the given time point in the stride cycle and *f* is the frequency. *P*_*xy*_(τ,*f*) is time-normalized time-dependent cross-spectrum between EVS and body kinematics, *P*_*xx*_(τ,*f*) is the time-normalized time-dependent auto-spectrum of the EVS signal, and *P*_*yy*_(τ,*f*) is the time-normalized time-dependent auto-spectrum of the ML linear acceleration.

#### 2.7.6 Head variability quantification

To quantify head movement variability during locomotion, we calculated the proportion of residual variance (*V*_*res*_) of the head linear accelerations and angular velocities, which has previously been used to predict the magnitude of vestibular-evoked balance responses during locomotion (MacNeilage and Glasauer, 2017; Dietrich et al., 2020). We computed *V*_*res*_ for the net head linear acceleration and net head angular velocity using the stride-normalized gravity-corrected and transformed mouthguard IMU data, as well as ML head linear acceleration and roll head angular velocity because the EVS evokes frontal plane virtual signals of head motion when looking forward during walking (Bent et al., 2004; Fitzpatrick et al., 2006).

### 2.8 Statistical analysis

We reported summary statistics as mean ± SD. To address our objectives of the study, we performed three interrelated analyses that examined the changes in coherence at the 52 and 78 steps/min conditions and sought to estimate the number of steps that are needed for consistent comparisons at these cadences.

For each participant, we determined time-dependent coherence to be significant if it crossed a 99% confidence limit (*CL* = 0.01) threshold which is equivalent to an alpha-level of 0.05 due to the two-dimensional (time and frequency) nature of the data (Blouin et al., 2011). Based on the number of strides (*n* = 250) used to calculate coherence, we determined the threshold was 0.018 (99% confidence limit). As spectral gain can only be interpreted when coherence is significant, we only considered and analyzed time-dependent gain when coherence was above the 0.018 threshold.

$$t\,h\,r\,e\,s\,h\,o\,l\,d=1-C\,L^{1/n}$$

To compare the changes in vestibular-evoked balance responses across time and frequency within the locomotor stride cycle, we compared the group-averaged results using a bootstrapping approach (Hannan et al., 2021). We randomly selected ten participants (with replacement) and extracted their cross and auto-spectra (*n* = 250 strides/participant) at both cadences. Then, we calculated the resampled group time-dependent coherences, gains, and spectral power outputs and calculated the differences between the two cadences. We repeated this process 10,000 times. At each time point and frequency, we sorted the data in an ascending order, and determined the 99% confidence interval by taking the 50 and 9950th data points. Points (across time and frequency) in the coherence, gain, and spectral power output differences where zero was not included in this 99% confidence interval were considered to be significantly different between cadences.

To further compare coherence between the two cadences, we also extracted peak coherence (across time and frequency) from each participant and cadence. We chose this approach because it has previously been used to compare the attenuation of vestibular-evoked balance responses between different cadences and speeds (Dietrich et al., 2020). We performed a one-sided paired student *T*-test (α = 0.05) (JASP Team, 2023) after correcting the non-normality of the coherence values with a Fisher transformation (Grosse and Brown, 2003).

To identify the appropriate number of strides needed to estimate vestibular-evoked responses from coherence estimates with our portable system, we compared coherence estimated from the full 250 strides and from the first *n* strides (in intervals of 1 stride, i.e., from 1 to 249 strides) of each participant using a bootstrapping protocol similar to the between-cadence comparisons described above. We randomly drew the same ten participants cross and auto-spectra at both the full number of strides (*n* = 250) and the first *n* strides from the data, with replacement. Then, we calculated the group time-dependent coherences, and their difference was taken between the 250 strides and the first *n* strides. We repeated this process 10,000 times and determined significant differences as above. We calculated the percentage of points in the time-frequency coherence representation exhibiting significant differences for each *n* strides and given that a confidence interval of 99% will result in a false positive rate of 1%, we expected the results to converge toward 1% when the null hypothesis (no difference in coherence) was true. To determine the number of strides where the percentage of different points increased with respect to the steady state percent difference (i.e., 229–249 strides) for each sensor and cadence, we adapted an algorithm proposed by Siegmund (2001). This adapted algorithm (percent error gradient detection) worked by moving from 249 strides to 1 stride to find the number of strides where the error rate increased, given the rate of change of the signal and baseline noise estimated from 249 to 229 strides. The algorithm parameter equivalent to frequency (strides^–1^) was set to 0.003.

To characterize changes to the estimates of head variability and predictability (*V*_*res*_), we extracted the overall mean across the stride cycle of each *V*_*res*_ measure for each participant and cadence. A one-sided paired student *T*-test (α = 0.05) (JASP Team, 2023) was then performed to determine changes in *V*_*res*_ between cadences. This approach has previously been used to compare changes in *V*_*res*_ across speeds and cadences (Dietrich et al., 2020).

## 3 Results

### 3.1 Gait characteristics

To measure phasic modulation of vestibular-evoked balance responses during locomotion, we first identified heel strikes and toe-offs using the IMUs placed on each ankle. The average stride duration was 2.31 ± 0.01 s for the 52 steps/min cadence and 1.54 ± 0.01 s for the 78 steps/min cadence. For the 52 and 78 steps/min cadences, left heel-strike occurred, respectively, at 50.4 ± 1.0% and 50.7 ± 1.0%, left toe-off at 21.5 ± 2.5% and 14.9 ± 1.9%, and right toe-off at 71.5 ± 2.6% and 64.7 ± 2.0% of the locomotor cycle.

### 3.2 Phasic modulation of the vestibular control of balance

**FIGURE 2:**
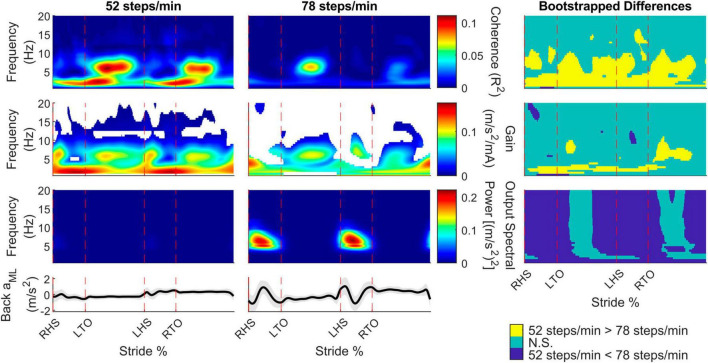
Participant pooled (*n* = 10) time-frequency analyses from the back mediolateral linear acceleration (*a*_*ML*_) for the 52 and 78 steps/min conditions. The stride-normalized time-dependent coherence (first row), time-dependent gain (second row), time-dependent spectral back *a*_*ML*_ power (third row), and average signal trace (fourth row; shaded area represents ± 1 standard deviation) are plotted. The columns categorize data from the 52 steps/minute condition (first column), the 78 steps/minute condition (second column), and the bootstrapped differences between cadences (third column). 52 > 78 steps/minute: the 52 steps/minute condition was significantly larger than the 78 steps/minute condition. N.S.: No significant difference between conditions. 52 < 78 steps/minute: the 78 steps/minute condition was significantly larger than the 52 steps/minute condition. RHS: right heel strike. LTO: left toe-off (18.2% of stride cycle). LHS: left heel strike (50.6%). RTO: right toe-off (68.1%), mA: milliAmperes.

**FIGURE 3:**
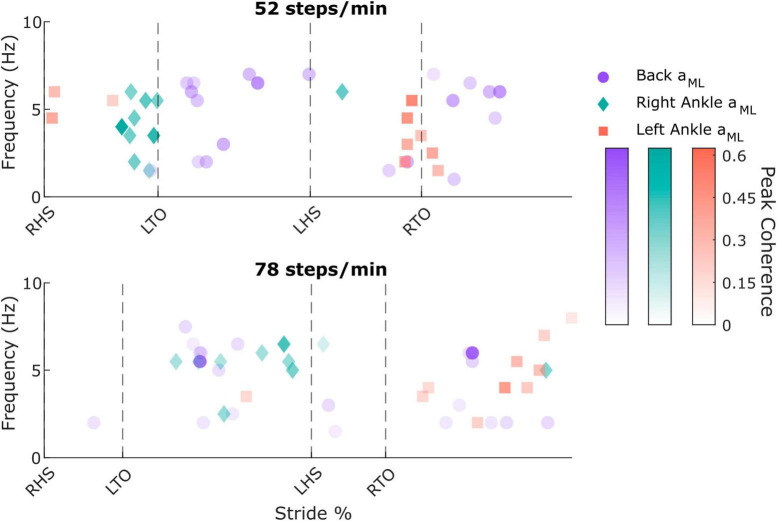
Individual participant peak coherence timing, frequency, and magnitude values from the back mediolateral linear acceleration (*a*_*ML*_; purple circles; two peaks were extracted for each participant), right ankle *a*_*ML*_ (green diamonds), and left ankle *a*_*ML*_ (red squares) in the 52 steps/minute **(top)** and 78 steps/minute **(bottom)** cadence conditions. RHS: right heel strike. LTO: left toe-off (52 steps/min: 21.5% of stride cycle, 78 steps/min: 14.9% of stride cycle). LHS: left heel strike (52 steps/min: 50.4%, 78 steps/min: 50.7%). RTO: right toe-off (52 steps/min: 71.5%, 78 steps/min: 64.7%).

To determine the presence of vestibular-evoked whole-body balance responses during real-world locomotion, we computed the time-dependent coherence between the applied stochastic EVS and the low back IMU ML linear acceleration (Figure 2). For all participants walking outside at both cadences, we observed phasic modulation of the vestibular-evoked responses through the stride cycle with coherence above the 99% significance threshold. During the 52 steps/min cadence, we observed peaks occurring after toe-offs (30.4 ± 6.0% and 75.4 ± 10.0% of the stride cycle) at 4.7 ± 2.2 Hz and 4.7 ± 2.3 Hz, respectively. At the 78 steps/min cadence, these peaks occurred at 25.3 ± 11.8% and 77.4 ± 13.7% of the stride cycle at 4.8 ± 1.9% Hz and 3.5 ± 1.8 Hz. Consequently, peak vestibular-evoked whole-body responses occurred during single support phases (Figure 3).

**FIGURE 4:**
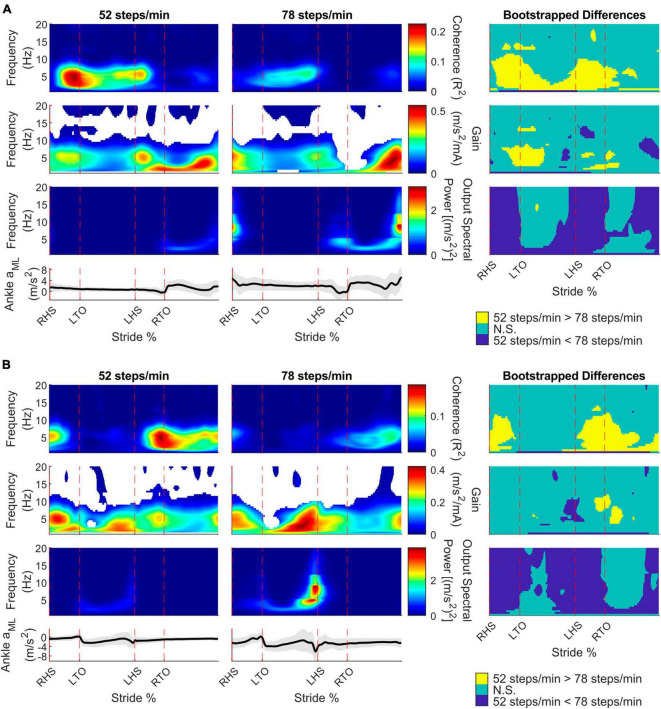
Participant pooled (*n* = 10) time-frequency analyses from the right ankle **(A)** and left ankle **(B)** mediolateral linear acceleration (*a*_*ML*_) for the 52 and 78 steps/min conditions. The stride-normalized time-dependent coherence (first row), time-dependent gain (second row), time-dependent spectral ankle *a*_*ML*_power (third row), and average signal trace (fourth row; shaded area represents ± 1 standard deviation) are plotted. The columns categorize data from the 52 steps/minute condition (first column), the 78 steps/minute condition (second column), and the bootstrapped differences between cadences (third column). 52 > 78 steps/minute: the 52 steps/minute condition was significantly larger than the 78 steps/minute condition. N.S.: No significant difference between conditions. 52 < 78 steps/minute: the 78 steps/minute condition was significantly larger than the 52 steps/minute condition. RHS: right heel strike. LTO: left toe-off (18.2% of stride cycle). LHS: left heel strike (50.6%). RTO: right toe-off (68.1%), mA: milliAmperes.

To characterize limb-specific vestibular-evoked balance responses, we also quantified coherence with the ML linear accelerations from the IMUs located on the right (Figure 4A) and left (Figure 4B) ankles. Again, all participants exhibited phasic modulation of the vestibular-evoked responses with time-dependent coherence reaching above the 99% significance threshold. For both ankles, peak coherence was mainly observed at the contralateral toe-off (right ankle: 9/10 participants, 18.1 ± 2.3%, 4 ± 1.6 Hz; left ankle: 7/10 participants, 70.8 ± 2.4% [20.4 ± 2.8% following left heel strike], 3.2 ± 1.4 Hz) during the 52 steps/min condition and just prior to the contralateral heel-strike in the 78 steps/min condition (right ankle: 9/10 participants, 39.4 ± 9.4%, 5.4 ± 1.2 Hz; left ankle: 9/10 participants, 87.1 ± 9.8% [36.5 ± 10.0% following left heel strike], 4.8 ± 1.8 Hz; Figure 3). Hence, peak vestibular-evoked lower limb responses occurred during the respective limb’s stance phase.

### 3.3 Vestibular control of balance decreases as cadence increases

To determine specific time and frequency dependent changes in the coherence, gain, and spectral output power between locomotor cadences, we performed bootstrapping analyses. For the back ML linear accelerations, coherence decreased throughout the stride as cadence increased for frequencies below 10 Hz. Gain did not change as much as coherence between cadences but also decreased for the faster cadence at similar regions as coherence. The back ML linear acceleration power increased for the 78 compared to 52 steps/min cadence, except during the middle of the single support phase (Figure 2, right column).

For the right and left ankle ML linear accelerations, coherence decreased below 10 Hz from the 52 to 78 steps/min at two main locomotor phases: contralateral toe-off and between the contralateral heel strike and ipsilateral toe-off. Similar to the back ML linear acceleration, gain mostly did not change, although we observed a decrease at the contralateral toe-off from 52 steps/min to 78 steps/min. ML ankle linear accelerations power generally increased with cadence, but areas of no significant change were seen following both toe-off events (Figure 4, right column).

**FIGURE 5:**
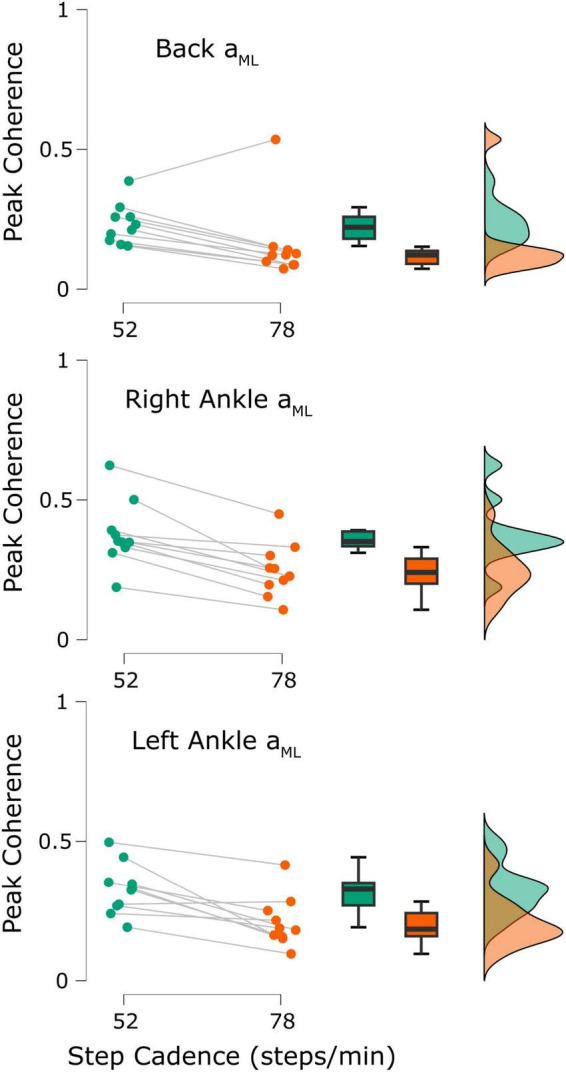
Participant peak coherence differences between the 52 steps/minute (green) and 78 steps/minute (orange) cadences (*n* = 10). Coherence was calculated between the applied stochastic vestibular stimulation and the back mediolateral linear acceleration (*a*_*ML*_; **top**), right ankle *a*_*ML*_ **(middle)**, and left ankle *a*_*ML*_ **(bottom)**. All differences were statistically significant (all *p*-values < 0.05).

To compare our results with previously reported observations, we also extracted each participant’s peak time-dependent coherence from both cadences. In 9/10 participants, peak coherence estimated in the low back ML acceleration decreased from the 52 to the 78 steps/min condition (52 steps/min = 0.23 ± 0.07, 78 steps/min = 0.16 ± 0.14, t_9_ = 2.37, *p* = 0.021; Figure 5, top; when removing the outlier participant: t_8_ = 9.03, *p* < 0.001). Similarly, coherence between stochastic EVS and ankle ML linear accelerations decreased for both the right ankle (10/10 participants; 52 steps/min = 0.38 ± 0.12, 78 steps/min = 0.25 ± 0.10, t_9_ = 5.91, *p* < 0.001; Figure 5, middle) and left ankle (9/10 participants; 52 steps/min = 0.33 ± 0.09, 78 steps/min = 0.21 ± 0.09, t_9_ = 4.37, *p* < 0.001; Figure 5, bottom) as cadence increased from 52 to 78 steps/min.

### 3.4 Minimum number of strides needed

**FIGURE 6:**
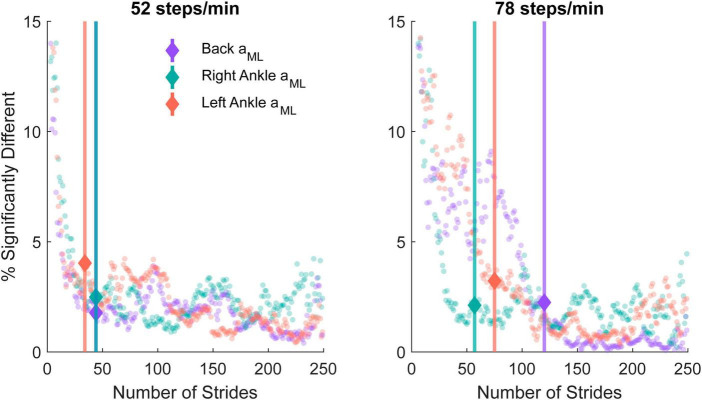
The percentage of significantly different time-frequency coordinates from the bootstrapped coherence difference between *n* and 250 strides in the 52 steps/minute **(left)** and 78 steps/minute **(right)** cadence conditions. A gradient detection algorithm was implemented to determine the number of strides at which the time-dependent coherence percentage differences increased beyond the expected 1% error rate for the back mediolateral linear acceleration (*a*_*ML*_; purple; 52 steps/min onset: 44 strides, 78 steps/min onset: 120 strides), right ankle *a*_*ML*_ (green; 52 steps/min onset: 44 strides, 78 steps/min onset: 57 strides), and left ankle *a*_*ML*_ (red; 52 steps/min onset: 34 strides, 78 steps/min onset: 75 strides). The minimal number of strides identified with the percentage error gradient detection algorithm is indicated with a marker and vertical line.

We quantified the lowest number of strides needed to estimate time-dependent coherence with minimal differences from 250 strides using a bootstrapping approach. For each *n* strides, we calculated the percentage of points in the time-frequency coherence representation exhibiting significant differences from coherence with 250 strides for both cadences and each sensor (Figure 6). For the 52 steps/min cadence, the percent error gradient detection algorithm determined that changes occurred at 44 strides for the low back ML acceleration as well as 44 and 34 strides for the right and left ankle ML accelerations, respectively. For the 78 steps/min cadence, the changes occurred at 120 strides for the low back, 57 for the right ankle, and 75 for the left ankle measures.

### 3.5 Head kinematics variability

**FIGURE 7:**
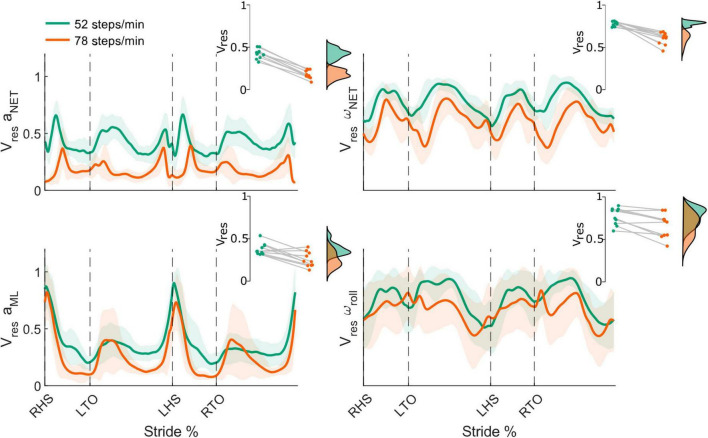
Stride-normalized head kinematic proportion of residual variance (*V*_*res*_) from the net head linear acceleration (*a*_*NET*_; **top left**) and head angular velocity (ω_*NET*_; **top right**), mediolateral head linear acceleration (*a*_*ML*_; **bottom left**), and roll head angular velocity (ω_*roll*_; **bottom right**) in the 52 (green) and 78 (orange) steps/minute conditions. Shaded areas represent ± 1 standard deviation. Insets illustrate the respective individual participant mean *V*_*res*_ for both conditions (green: 52 steps/minute and orange: 78 steps/minute). All differences were statistically significant (all *p*-values < 0.05). RHS: right heel strike. LTO: left toe-off (18.2% of stride cycle). LHS: left heel strike (50.6%). RTO: right toe-off (68.1%).

Using data from the mouthguard instrumented IMU, we characterized head kinematics variability during real-world locomotion using the net and frontal plane *V*_*res*_. All linear and angular measures of head movement variability were modulated throughout the stride cycle, with peaks occurring around heel strikes and between ipsilateral leg toe-off and heel strike (Figure 7).

From the 52 to 78 steps/min cadence, the mean net *V*_*res*_ significantly decreased for both the net head linear acceleration (52 steps/min = 0.42 ± 0.06, 78 steps/min = 0.18 ± 0.05, t_9_ = 17.84, *p* < 0.001) and net angular velocity (52 steps/min = 0.78 ± 0.03, 78 steps/min = 0.61 ± 0.07, t_9_ = 7.12, *p* < 0.001) (Figure 7, top row). We also observed significant decreases for the mean ML linear acceleration *V*_*res*_ (52 steps/min = 0.38 ± 0.07, 78 steps/min = 0.26 ± 0.09, t_9_ = 4.02, *p* = 0.002) and the mean roll angular velocity *V*_*res*_ (52 steps/min = 0.77 ± 0.10, 78 steps/min = 0.66 ± 0.14, t_9_ = 3.86, *p* = 0.002) as locomotor cadence increased (Figure 7, bottom row).

## 4 Discussion

In this study, we developed a portable, IMU-based system to measure vestibular-evoked balance responses during real-world locomotion in healthy participants. Using this system, we applied EVS and measured body segment kinematics to characterize movements and balance responses in everyday environments. All participants exhibited phasic modulation of vestibular-evoked whole-body and lower limb responses during the locomotor cycle. As hypothesized, the magnitude of the vestibular-evoked balance responses decreased with increasing locomotor speed and step cadence. Furthermore, we determined that only 34–120 strides are needed to characterize these responses at the examined cadences. These results confirm previous observations and demonstrate that our portable system can be a useful tool to uncover neuroscience principles in the wild.

### 4.1 Kinematic measures can characterize whole-body and limb specific vestibular-evoked responses

By computing the coherence between a commonly used stochastic EVS signal and the ML linear acceleration from IMUs positioned on the low back and both ankles, we found vestibular-evoked balance responses above a 99% confidence interval in all participants for both the 52 and 78 steps/min cadence conditions. This technique has previously been used to compare an applied vestibular error-signal with muscle activity, ground reaction forces and moments, or body kinematics in quiet standing (Dakin et al., 2007; Horslen et al., 2014; de Melker Worms et al., 2017) and walking conditions (Bent et al., 2004; Iles et al., 2007; Blouin et al., 2011; Dietrich et al., 2020). Here, we extended these findings by demonstrating that it is possible to use linear accelerations measured from wearable IMUs to characterize vestibular-evoked responses.

Furthermore, we observed phasic modulation of vestibular-evoked balance responses across the stride cycle. When representing the whole-body centre of mass ML linear acceleration using an IMU placed on the low back, the vestibular-evoked whole-body balance responses peaked between toe-off and heel strike for each limb. Previous researchers using force platforms have observed responses at similar times in the stride cycle (Dietrich et al., 2020; Magnani et al., 2021) likely because the whole-body ML accelerations we quantified using an IMU are related to ground reaction forces and centre of pressure displacement measured from an instrumented treadmill. Additionally, we also observed limb-specific modulation of the vestibular-evoked responses. The magnitude of the coherence between EVS and the ML linear acceleration of both ankles was largest during the ipsilateral limb stance phase. Previous studies have observed similar modulation, with vestibular-evoked responses measured from lower limb muscle activity strongest during the stance phase (Iles et al., 2007; Blouin et al., 2011; Dakin et al., 2013). This suggests that the vestibular influence is greatest at this point in the stride cycle because it is when the limb and muscles are most actively involved in balance control. During the 52 steps/min condition, we observed that the peak ankle responses occurred at toe-off, slightly prior to the peak low back responses observed at midstance. In the 78 steps/min condition, the peak ankle and low back responses both occurred during midstance.

These results indicate that IMUs are a viable alternative to measure vestibular-evoked responses during locomotion, which will provide greater flexibility to future research on vestibular balance control in natural environments.

### 4.2 Vestibular control of balance decreases with faster cadence and speed during real-world locomotion

Both the bootstrapped coherence differences and extracted peak coherences showed a clear decrease from the 52 to 78 steps/min cadence for all sensor measures. The peak coherences decreased by ∼34–38% between the cadences. Previous studies have shown similar changes in peak coherence between the same cadences and speeds used in the current study, with a decrease of ∼47% observed via centre of pressure measurements (Dietrich et al., 2020) and reductions of 17–31% peak coherence in lower limb muscle activity (Dakin et al., 2013).

Using the bootstrapping approach, we observed concurrent decreases in gain between cadences at regions similar to the observed decreases in coherence for the low back ML acceleration and ankle ML accelerations. Both coherence and gain have been used to infer vestibular balance control and have been shown to decrease concurrently during locomotion (Blouin et al., 2011; Forbes et al., 2017; Magnani et al., 2021). As gain represents the scaling between the applied EVS and the body kinematics, this decrease supports the notion that the decreased coherence is due to reduced vestibular input, rather than related to an increasing magnitude of movement at faster speeds (Dakin et al., 2013). The signal power at these regions did not change according to the bootstrapping analyses, providing further evidence that the decreases in coherence and gain are due to lower vestibular input rather than added noise from increased movement output spectral power (Thomas, 2015).

For the single participant where coherence between EVS and the low back ML acceleration increased with the faster cadence, we noted that the participant adopted a wider step width at the 52 steps/min cadence to compensate for the balance perturbations evoked by EVS. Given that previous work using ground reaction forces has observed a decrease in vestibular influence when step width increased, this likely explains this deviation from the other participants’ responses (Magnani et al., 2021). However, the coherence from the ankle IMUs did not increase with the low back at the faster cadence, suggesting that while whole-body balance responses to EVS are altered with increased step width, the individual lower limbs remained similarly influenced by vestibular error signals despite the stance width effects on whole-body motion.

Currently, the only proposed model explaining the decrease in vestibular-evoked balance responses during faster walking suggests that this is driven by the ratio of motor uncertainty to vestibular uncertainty, which is quantified with the *V*_*res*_ (MacNeilage and Glasauer, 2017). Our results support this hypothesis and previous observations (Dietrich et al., 2020), given that both the coherence and *V*_*res*_ decreased with faster step cadences for the locomotor parameters explored here. Alternatively, the vestibular responses could be influenced by body stability demands. This explanation is congruent with the observed effects of step width on vestibular balance responses, in the single participant mentioned above and previous work (Magnani et al., 2021), and individual limb response adaptations during split-belt treadmill walking (Forbes et al., 2017). The changes in the timing of vestibular-evoked responses across cadences could also be related to stability, but more work is needed to examine this. Future research using the experimental set-up validated in this study will be able to characterize head kinematics and vestibular-evoked balance responses in a wide variety of environments to determine the mechanisms that modulate the vestibular control of balance during locomotion.

### 4.3 Number of strides needed to estimate vestibular-evoked responses

We also determined the number of strides needed to appropriately characterize vestibular-evoked balance responses with minimal differences from estimates obtained with 250 strides (Figure 6). For the three sensor measures we evaluated, we found that 34–44 strides were needed at 52 steps/min and 57–120 strides were needed at 78 steps/min. Increasing cadence results in a lower coherence magnitude, which in turn leads to a lower signal-to-noise ratio. We propose this lower signal to noise ratio likely explains why more strides are needed to characterize vestibular-evoked balance responses as the cadence increases.

Consequently, although we found that no more than 120 strides were needed for the cadences and sensor measures used in the present study, it is likely that the minimum number of strides would increase for faster cadences or speeds. This minimum number of strides is also lower than the previously recommended 250 strides (Blouin et al., 2011), a study in which the authors used electromyography to characterize the vestibular-evoked responses. It is possible that surface electromyographic signals recorded from lower limb muscles may provide noisier estimates of vestibular-evoked responses. The fewer strides needed to characterize vestibular-evoked responses with kinematic than electromyographic measures can inform more efficient data collection in future studies although a direct comparison between recording methods is needed. It is important to also note that more than 250 strides may be needed to confirm that our lower estimates in the minimal required number of strides. The convergence of the percentage of significantly different points toward the expected 1% error rate after 120 strides for all measures and cadences, however, supports our findings. This suggests that adding more strides may yield limited benefits.

### 4.4 Head kinematics

To quantify head movement variability and its potential link to vestibular-evoked balance responses, we calculated *V*_*res*_ of the net head linear acceleration, net head angular velocity, ML head linear acceleration, and roll head angular velocity using the mouthguard-instrumented IMU. Similar to previous studies, mean *V*_*res*_ decreased at faster speeds and cadences (MacNeilage and Glasauer, 2017; Dietrich et al., 2020). Given that *V*_*res*_ is the ratio of residual variability (assumed to be motor noise) to total variability (assumed to be sensory/vestibular noise), this suggests motor uncertainty decreased relative to vestibular uncertainty, a possible explanation for the reduced vestibular-evoked balance responses.

We chose to calculate *V*_*res*_ from the head kinematics because it has previously been used to predict vestibular-evoked balance responses during locomotion; however, there are other measures of head stability that could also be characterized with our portable set-up (Pozzo et al., 1990; Menz et al., 2003; Latt et al., 2007). Furthermore, by using a mouthguard to rigidly attach the IMU to the participant’s skull, we limited the influence of skin artefact on our signals (Wu et al., 2016), thus providing an accurate representation of naturalistic head kinematics during locomotion.

### 4.5 Limitations

The main limitation of this study is sensor placement. Due to the constraints of the portable system, we could only collect from four IMUs simultaneously. While we observed significant vestibular-evoked balance responses in all participants from the low back and ankle sensors, other IMU placements could be explored. Notably, de Melker Worms et al. (2017) observed the strongest lower limb vestibular responses from the knee kinematics, suggesting that this may be an ideal placement for measuring vestibular responses. However, we chose the ankles as the location for the IMUs to better identify gait events as well as possibly characterize vestibular-evoked balance responses.

Another limitation was the restriction of natural head movements. While we presented head kinematic data as a possible outcome measure from this novel portable system, the interpretation of these data may be restricted because we instructed participants to maintain a head pitch with Reid’s plane ∼17–19° above the horizontal. This was done to maximize the effect of the EVS as is standard in studies using electrical vestibular stimulation (Cathers et al., 2005; Day and Fitzpatrick, 2005b; Mian et al., 2010; Reynolds, 2011); however, future studies characterizing naturalistic head movements and optic flow should allow participants to adopt more natural head positions.

Our data collections occurred in outdoor walking scenarios that were linear in nature and on level ground. While this provided a more dynamic environment than laboratory settings, it is still a semi-controlled environment when compared to normal daily walking. Hence, it is still unclear if the procedures presented in this study can be used in more free-living scenarios with complex walking behaviours. Future studies are needed to explore these more complex scenarios. Finally, the study population comprised only healthy, young adults. This ensured our results were free of confounding factors (age, musculoskeletal disease, and balance impairments) that could affect locomotion and its multisensory feedback control. Characterizing vestibular influences on dynamic balance in clinical populations could reveal how balance is adapted in real-world conditions.

### 4.6 Conclusion

In summary, we developed a fully portable system to apply electrical vestibular stimulation and measure head and body kinematics using inertial measurement units. We characterized the vestibular-evoked balance responses during real-world locomotion by quantifying the relationship between the applied vestibular stimulus and the mediolateral linear acceleration from sensors located on the low back and both ankles. Similar to in-laboratory studies, we observed phase- and speed/cadence-dependence of the vestibular control of balance during locomotion, with the responses decreasing with increasing speed and cadence. Here, we have extended these results to IMU kinematics recorded during locomotion in the real-world. These results demonstrate that the vestibular control of balance during locomotion can be accurately characterized using a fully portable system in semi-controlled outdoor environments. The present research represents a critical first step to understand the vestibular control of balance in the wild and reveal the neural mechanisms driving vestibular modulations in naturalistic and variable movements experienced in our everyday life.

## Data availability statement

The datasets presented in this study can be found in online repositories. The names of the repository/repositories and accession number(s) can be found below: https://doi.org/10.5683/SP3/0MDZFR.

## Ethics statement

The studies involving humans were approved by the University of British Columbia Clinical Research Ethics Board (H22-01776). The studies were conducted in accordance with the local legislation and institutional requirements. The participants provided their written informed consent to participate in this study.

## Author contributions

LF: Conceptualization, Formal analysis, Investigation, Methodology, Software, Writing – original draft, Writing – review & editing. JC: Methodology, Writing – review & editing. J-SB: Conceptualization, Funding acquisition, Methodology, Resources, Supervision, Writing – review & editing.
